# Spatial Heterogeneity of Enteric Fever in 2 Diverse Communities in Nepal

**DOI:** 10.1093/cid/ciaa1319

**Published:** 2020-12-01

**Authors:** Dipesh Tamrakar, Krista Vaidya, Alexander T Yu, Kristen Aiemjoy, Shiva Ram Naga, Yanjia Cao, Caryn Bern, Rajeev Shrestha, Biraj M Karmacharya, Sailesh Pradhan, Farah Naz Qamar, Samir Saha, Kashmira Date, Ashley T Longley, Caitlin Hemlock, Stephen Luby, Denise O Garrett, Isaac I Bogoch, Jason R Andrews

**Affiliations:** 1 Dhulikhel Hospital, Kathmandu University Hospital, Kavrepalanchok, Nepal; 2 Division of Infectious Diseases and Geographic Medicine, School of Medicine, Stanford University, Stanford, California, USA; 3 Department of Epidemiology and Biostatistics, University of California, San Francisco, San Francisco, California, USA; 4 Kathmandu Medical College and Teaching Hospital, Kathmandu, Nepal; 5 Department of Pediatrics and Child Health, Aga Khan University, Karachi, Pakistan; 6 Child Health Research Foundation, Department of Microbiology, Dhaka Shishu (Children’s) Hospital, Dhaka, Bangladesh; 7 Global Immunization Division, Centers for Disease Control and Prevention, Atlanta, Georgia, USA; 8 National Foundation for the Centers for Disease Control and Prevention, Atlanta, Georgia, USA; 9 Applied Epidemiology, Sabin Vaccine Institute, Washington, DC, USA; 10 Department of Medicine, University of Toronto, Toronto, Canada

**Keywords:** typhoid, enteric fever, *Salmonella*, Nepal, geospatial

## Abstract

**Background:**

Typhoid fever is endemic in the urban Kathmandu Valley of Nepal; however, there have been no population-based studies of typhoid outside of this community in the past 3 decades. Whether typhoid immunization should be prioritized in periurban and rural communities has been unclear.

**Methods:**

We performed population-based surveillance for enteric fever in 1 urban catchment (Kathmandu) and 1 periurban and rural catchment (Kavrepalanchok) as part of the Surveillance for Enteric Fever in Asia Project (SEAP). We recruited individuals presenting to outpatient and emergency departments at 2 study hospitals with suspected enteric fever and performed blood cultures. Additionally, we conducted a household survey in each catchment area to characterize care seeking for febrile illness. We evaluated spatial heterogeneity in febrile illness, care seeking, and enteric fever incidence.

**Results:**

Between September 2016 and September 2019, we enrolled 5736 participants with suspected enteric fever at 2 study hospitals. Among these, 304 (5.3%) were culture positive for *Salmonella* Typhi (249 [81.9%]) or Paratyphi A (55 [18.1%]). Adjusted typhoid incidence in Kathmandu was 484 per 100 000 person-years and in Kavrepalanchok was 615 per 100 000 person-years. While all geographic areas for which estimates could be made had incidence >200 per 100 000 person-years, we observed spatial heterogeneity with up to 10-fold variation in incidence between communities.

**Conclusions:**

In urban, periurban, and rural communities in and around Kathmandu, we measured a high but heterogenous incidence of typhoid. These findings provide some support for the introduction of conjugate vaccines in Nepal, including outside urban areas, alongside other measures to prevent enteric fever.

Enteric fever, caused by *Salmonella enterica* subspecies *enterica* serotypes Typhi and Paratyphi A, B, and C, is among the leading causes of invasive bacterial infection in South Asia [1, 2]. The World Health Organization has recommended the introduction of typhoid conjugate vaccine (TCV) in settings with high typhoid incidence or high rates of antimicrobial-resistant typhoid [3]. Health policy makers in Nepal, as in many other countries, have been faced with deciding whether and where to deploy TCV vaccinations with limited typhoid incidence data outside of focused areas. As part of the Surveillance of Enteric Fever in Asia Project (SEAP), we sought to understand the population incidence of enteric fever in diverse communities in Nepal.

Studies over the past 2 decades have revealed a high degree of endemicity in the Kathmandu Valley, the largest urban center in the country [4, 5]. However, the majority of typhoid studies conducted over the past 20 years have been in a single urban center in the Kathmandu Valley, Lalitpur [4–7], which has consistently shown a high burden of typhoid in children (428 cases per 100 000 person-years [PY] in the control arm of the recent vaccine trial). There have been no population-based typhoid studies in Nepal outside of this community since the 1980s, when the Vi polysaccharide vaccine was first evaluated [8]. Even elsewhere within the Kathmandu Valley, the only available data have been through retrospective analysis of passively collected culture data, which do not provide population-level incidence estimates [9].

As 80% of the population of Nepal lives outside of urban areas [10], there is need for typhoid incidence estimates from periurban and rural communities to determine whether the pattern of enteric fever occurrence warrants introduction of TCVs nationwide. While data on clinically diagnosed enteric fever cases are collected through a national reporting system, such diagnoses are not based on culture confirmation, and a recent study in the country found poor correlation between clinically reported typhoid and culture-confirmed disease [11]. To address this knowledge gap, we undertook prospective, population-based surveillance using blood cultures in urban, periurban, and rural communities in Kathmandu and Kavrepalanchok, Nepal. Here, we report on spatial heterogeneity in enteric fever symptoms, care-seeking, and culture-confirmed typhoid and paratyphoid disease in these communities.

## METHODS

### Study Design

SEAP was a prospective, population-based study conducted in communities in Nepal, Bangladesh, and Pakistan between September 2016 and September 2019. The study utilized a “hybrid surveillance” approach to estimate incidence [12], combining hospital-based surveillance to identify enteric fever cases and a community-based healthcare utilization survey to estimate the proportion of enteric fever cases captured by surveillance hospitals. The clinical characteristics and outcomes were reported separately [13, 14]. Here, we report on heterogeneity in healthcare seeking and enteric fever incidence in Nepal.

### Study Site and Population

This study was conducted in 2 settings in Nepal, defined by the catchment area for the 2 main SEAP surveillance hospitals: Kathmandu Medical College and Teaching Hospital in Kathmandu Metropolitan City and Dhulikhel Hospital in Kavrepalanchok District, which is approximately 30 km east of Kathmandu. Kathmandu is the capital of Nepal and has a dense, urban population of >20 000 people per square kilometer. The Kathmandu catchment area for this study was identified based on review of the home addresses of 100 consecutive patients meeting SEAP clinical enrollment criteria (fever on at least 3 days within the past 7 days). We selected 9 wards (6, 7, 8, 9, 10, 32, 33, 34, and 35) within Kathmandu, which accounted for the home addresses of >60% of patients meeting the study definition. In Kavrepalanchok, which has periurban and rural populations, we identified the home address of the past 100 patients with culture-confirmed enteric fever diagnosed at Dhulikhel Hospital and selected 4 municipalities, which accounted for >60% of cases. These municipalities consisted of Dhulikhel (12.1 km^2^; density: 1066 persons/km^2^), Banepa (5.6 km^2^; 4050 persons/km^2^), Paanchkhal (19.1 km^2^; 387 persons/km^2^), and Panauti (31.7 km^2^; 723 persons/km^2^). We utilized administrative geographical units (eg, wards, municipalities) rather than physical boundaries to facilitate the determination of whether participants arriving at the hospital lived within the catchment area. Because the most recent available population census was 8 years old, and there had been substantial population shifts over the interim (particularly following the 2015 earthquake), we estimated the population from the healthcare utilization survey as described below.

### Study Procedures and Definitions

#### Clinical Surveillance

We prospectively enrolled participants from the 2 main SEAP surveillance hospitals. At each hospital, we screened all patients presenting to the outpatient departments (adult and pediatric) and emergency department for history of fever. Those reporting fever on at least 3 consecutive days within the past 7 days who resided within the predefined catchment area were invited to participate in the study. In the inpatient department, we recruited all participants who were suspected of enteric fever by clinicians. There were no age restrictions for enrollment. Participants were recruited 6 days per week (Sunday through Friday); those who sought care in the emergency room or inpatient department overnight or on Saturdays were recruited the following day if still present. After obtaining informed consent, we administered a standardized questionnaire to ascertain demographic and clinical information. We collected 2–10 mL of peripheral blood, which were inoculated into BACTEC Aerobic or Peds Plus bottles and incubated using a BACTEC automated system for up to 5 days. Bottles showing growth were subcultured on sheep blood agar and MacConkey agar, and biochemical and antisera testing was performed to identify isolates. We defined typhoid and paratyphoid cases as individuals with positive blood cultures for *Salmonella* Typhi or *Salmonella* Paratyphi A, B, or C. Additionally, we established a network of 7 microbiology laboratories in Kathmandu (Bir Hospital, Helping Hands Community Hospital, Nepal Medical College, Kathmandu Model Hospital, Alka Hospital, Kanti Children’s Hospital, and Nepal Police Hospital). We contacted all patients identified at these laboratories with culture-confirmed enteric fever cases for enrollment in the study.

#### Healthcare Utilization Survey

We conducted a household-based healthcare utilization survey in both catchment areas [15, 16]. In brief, we used grid-based random sampling to select geographic clusters, from which all households were approached. A standardized questionnaire was administered to the head of all consenting households, with the primary objective of determining the proportion of individuals with a typhoid-like illness (fever ≥3 days) who sought care at the 2 surveillance hospitals. We inquired about any healthcare seeking for fever ≥3 days that occurred within the past 8 weeks, as well as any hospitalization for fever within the past year, reasoning that recall accuracy would be longer for hospitalization.

### Analytic Approach

We characterized enteric fever cases by demographic variables, reporting median and interquartile range (IQR) for continuous variables and proportions for dichotomous variables. For the healthcare utilization survey, we used mixed effects logistic regression models with random effects for cluster to estimate the proportion seeking care at the study site. We performed spatial interpolation to estimate local density of fever and care seeking using an inverse distance weighted model. We assessed spatial risk factors for fever and care seeking using logistic regression, and investigated the effects of community water sources and wealth. We defined improved water sources as municipally supplied water or that purchased from a vendor, and unimproved water sources as surface waters, rainwater, public taps, or groundwater. We created a household wealth index using principal components analysis using the following assets: electricity, ownership of radio, television, landline telephone, mobile phone, computer, watch, bicycle, motorcycle, car, and bank account. We classified administrative areas (wards and municipalities) by population density as urban (≥5000 persons/km^2^), periurban (1000–4999 persons/km^2^), or rural (<1000 persons/km^2^).

We used OpenStreetMap (accessed in October 2019 using QGIS 3.8) to build a street network and estimated road distance from household to surveillance hospital using Origin-Destination Matrix in ArcGIS 10.7.1 (Esri, Redlands, California). To assess the relationship between distance and care seeking, we fit both generalized linear models and generalized additive models, both with logistic link functions, and compared models by Akaike Information Criteria and χ ^2^ goodness of fit.

The primary objective of this study was to estimate incidence of typhoid and paratyphoid fever in various geographic areas. We have previously described the overall analytic approach used for this study [12]. We first estimated the crude, culture-confirmed typhoid and paratyphoid incidence by dividing all culture-confirmed cases by the catchment population and duration of the study. The catchment population and population of each administrative area was estimated by dividing the measured population from the healthcare utilization survey by the cluster sampling fraction and the response rate. In the crude estimates, we included all cases who resided in the catchment area that were identified through surveillance at the hospital and laboratory network sites.

We then undertook several adjustments to estimate the true incidence of typhoid and paratyphoid. For the adjusted estimates, we excluded cases enrolled from the laboratory network, as systematic active surveillance was not undertaken at those facilities. First, we adjusted for blood culture sensitivity, estimating it to be 59% (95% confidence interval [CI], 54%–64%) [17], and dividing our crude estimate by this number. Second, we adjusted to account for eligible patients who were missed by surveillance, either because they presented during evenings, weekends, or when the study staff were unavailable, consented to enrollment but did not have a blood culture obtained, or because they were approached but declined to participate. We divided our estimate by the proportion of cases that were captured by the surveillance study. Finally, we adjusted for individuals who sought care at sites other than the surveillance sites by dividing by the proportion seeking care at the study site, stratified by each age group and geographical unit. We propagated uncertainty in culture sensitivity and care seeking through Monte Carlo sampling from distributions estimated for each of these parameters and generated 95% credible intervals for incidence estimates.

All analyses and geo-visualization were performed using ArcGIS 10.7.1 (Esri) and R software.

### Ethics Statement

All participants provided informed consent. For participants under age 18, a parent or guardian provided informed consent, and those 7–17 years of age provided assent. The study was approved by the institutional review boards at Kathmandu University School of Medical Sciences, Stanford University, the Nepal Health Research Council, and through local ethics boards at participating hospitals.

## RESULTS

Between September 2016 and September 2019, we enrolled 5667 participants with suspected enteric fever at the 2 study hospitals (Dhulikhel Hospital: 2434; Kathmandu Medical College Hospital: 3233). Among these, 4827 (85.2%) were enrolled from outpatient or emergency departments, and the remaining 841 were enrolled from inpatient wards. Overall, 304 (5.4%) of these patients were culture positive for enteric fever. Additionally, we identified 1296 cases from the laboratory network sites. Among all culture-confirmed cases, 1366 (85.4%) were *Salmonella* Typhi and 234 (14.6%) were *Salmonella* Paratyphi A. The majority (58.9%) of cases occurred among males. The median age of *S.* Paratyphi A patients (21 [IQR, 17–26] years) was slightly higher than that of Typhi patients (19.5 [IQR, 15–24] years; *P* = .0003). Approximately one-quarter of typhoid cases (369/1369 [27.0%]) occurred among children <16 years of age, and more than half (720/1369 [52.6%]) occurred among individuals aged 16–25 years. Age distribution of typhoid cases did not differ between Kathmandu and Kavrepalanchok (*P* = .414).

**Figure 1.**
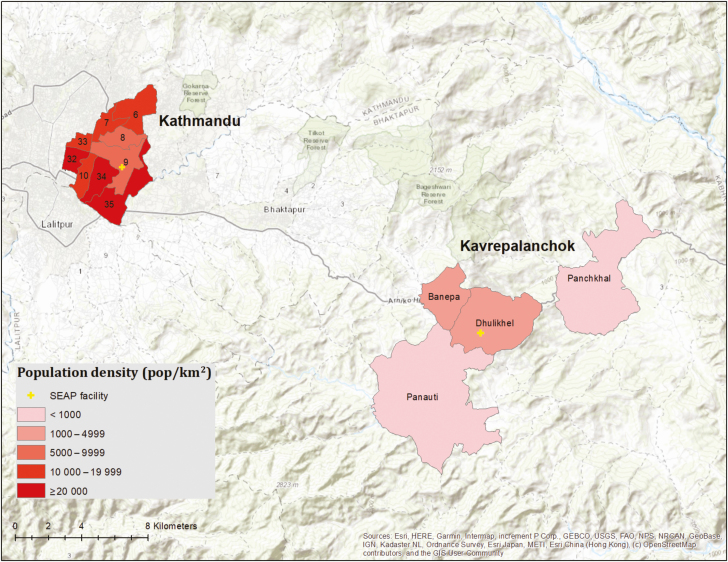
Population density in the Kathmandu wards and Kavrepalanchok municipalities comprising the study catchment area. Yellow crosses denote the location of study surveillance hospitals. Abbreviation: SEAP, Surveillance for Enteric Fever in Asia Project.

We conducted healthcare utilization surveys continuously for 24 months, from January 2017 through December 2018. We enrolled 16 744 households in Kathmandu (covering 50 039 participants) and 8729 households in Kavrepalanchok (34 041 participants) (Figure 1). Among those in Kavrepalanchok, 59% were in periurban areas and 41% were in rural areas. The most common drinking water source in urban areas was delivery by truck (38.0%) followed by piped into house (23.9%). In periurban areas, the most common drinking water source was water piped into the household (61%), and in rural areas, surface waters were the most common source of drinking water (38.6%). Household toilet ownership was high (>95%) in all areas.

**Figure 2.**
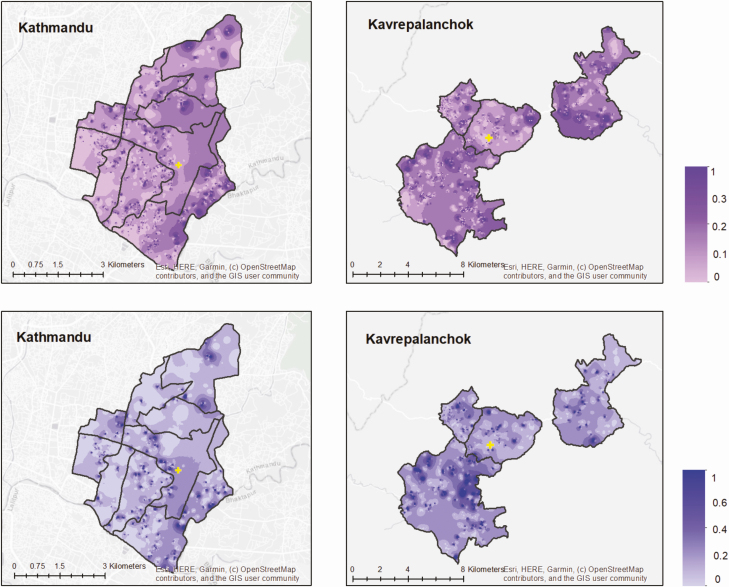
Proportion of individuals with fever in the past 8 weeks (top) and hospitalized for fever in the past year (bottom) for Kathmandu and Kavrepalanchok. Yellow crosses denote the location of study surveillance hospitals.

Among enrolled households, 919 (5.4%) in Kathmandu and 1353 (15.5%) in Kavrepalanchok, respectively, reported at least 1 member with febrile illness within the past 8 weeks, and 188 (1.1%) and 324 (3.7%) reported 1 member with hospitalization for fever within the past year. We found spatial heterogeneity in the proportion of households reporting febrile illness in the past 8 weeks (Figure 2). Households in communities lacking improved water (odds ratio [OR], 2.26; *P* < .0001) and lower wealth quintiles (OR, 1.06 per quintile; *P* = .008) were more likely to report a member having fever in the past 8 weeks.

**Figure 3.**
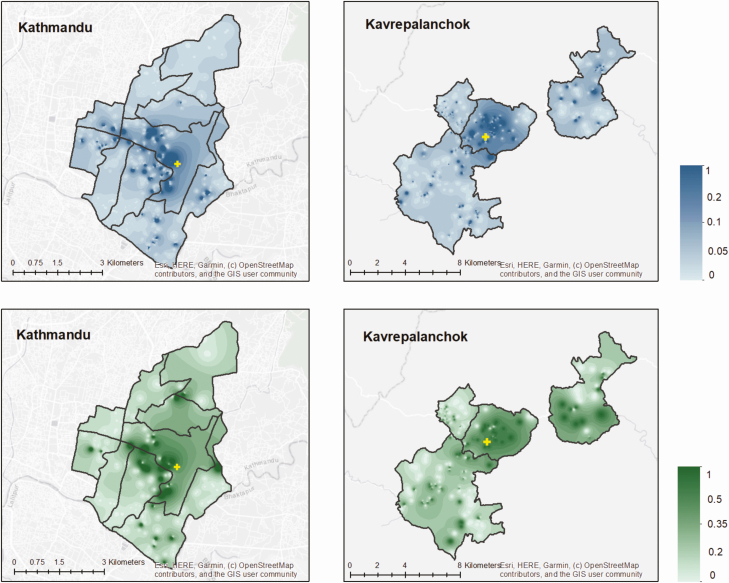
Proportion of individuals with fever in past 8 weeks (top) or hospitalized in past month (bottom) who sought care at the study hospitals in Kathmandu and Kavrepalanchok. Yellow crosses denote the location of study surveillance hospitals.

**Figure 4.**
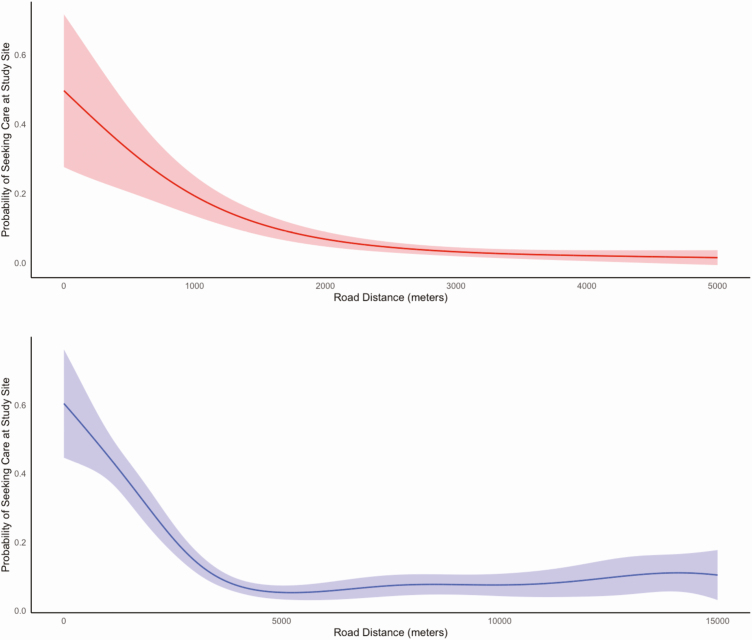
Probability of seeking care at study site as a function of road distance between household and the study site for Kathmandu (top) and Kavrepalanchok (bottom).

In Kathmandu, 6.6% of individuals with fever in the past week and 4.2% of individuals hospitalized for fever in the past year sought care at the study site. In Kavrepalanchok, 13.3% of individuals with fever and 12.6% of individuals hospitalized for fever sought care at the study site. We noted substantial heterogeneity across each catchment area in the proportion seeking care at the study site (Figure 3). We tested how geodesic distance and road length between households and surveillance hospitals affected probability of seeking care at that study site. We found that road length was a better predictor of care seeking than geodesic distance for both catchment areas (*P* < .0001 for model comparisons), and that the relationship was nonlinear (Figure 4). In both communities, participants in the first quintile of road distance were far more likely to seek care at the study site compared with those in the top 3 quintiles for distance (Kathmandu: 17.7% vs 3.1%; *P* < .0001; Kavrepalanchok: 34.2% vs 7.0%; *P* < .0001).

**Figure 5.**
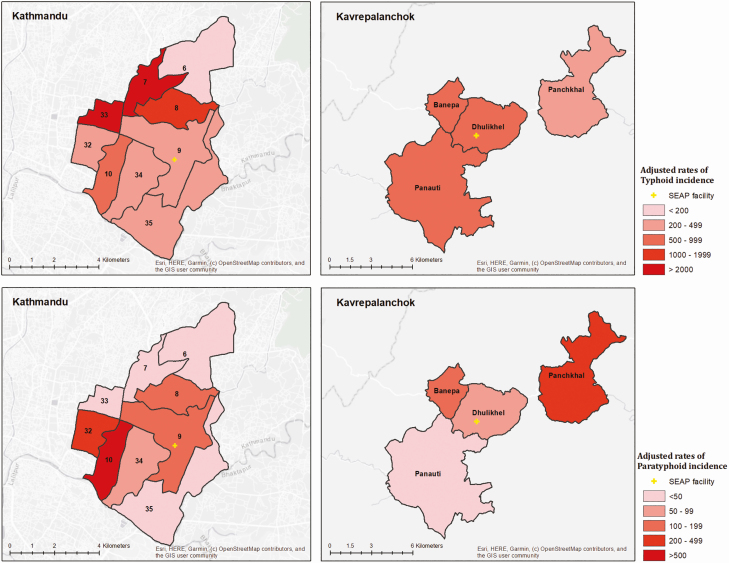
Incidence (cases per 100 000 person-years) of typhoid (*A*) and paratyphoid (*B*) by ward in Kathmandu and by municipality in Kavrepalanchok. Yellow crosses denote the location of study surveillance hospitals. Abbreviation: SEAP, Surveillance for Enteric Fever in Asia Project.

The crude incidence of blood culture–confirmed typhoid and paratyphoid fever was 31 (95% CI, 26–37) and 6 (95% CI, 4–9) cases per 100 000 PY, respectively, in Kathmandu and 36 (95% CI, 24–51) and 7 (95% CI, 3–16) cases per 100 000 PY, respectively, in Kavrepalanchok. After adjusting for culture sensitivity, enrollment capture, and care-seeking, we found a high incidence of typhoid in both communities (484 [95% CI, 384–612] per 100 000 PY in Kathmandu; 615 [95% CI, 527–721] per 100 000 PY in Kavrepalanchok). In 1 ward of Kathmandu (ward 6), which was furthest from the surveillance hospital, only 25 participants were enrolled in clinical surveillance and none had typhoid. For the remaining 8 wards in Kathmandu and 4 municipalities in Kavrepalanchok, estimated typhoid incidence was >200 per 100 000 PY (Figure 5). The highest incidence was in wards 7 and 33 (2510 per 100 000 PY and 2661 per 100 000 PY), and the lowest incidence occurred in ward 34 (223 per 100 000 PY) and Panauti municipality (309 per 100 000 PY). Paratyphoid incidence was 117 (95% CI, 93–148) per 100 000 PY in Kathmandu and 105 (95% CI, 90–123) cases per 100 000 PY in Kavrepalanchok, with substantial geographic heterogeneity ranging between 642 per 100 000 ward 10 to 0 per 100 000 in ward 33 of Kathmandu.

## DISCUSSION

In this population-based study in 2 areas in Nepal, we found an overall high incidence of enteric fever, >200 cases per 100 000 PY in all geographic areas for which we were able to make estimates. We also noted substantial heterogeneity in incidence, ranging from just over 200 cases per 100 000 PY to >2000 cases per 100 000 PY. Incidence of typhoid fever was 4–6 times greater than that of paratyphoid fever, which was an average of 80–100 cases per 100 000 PY. While individuals living far from study sites were less likely to seek care for febrile illness, after adjusting for this we did not detect a consistent relationship between population density and typhoid incidence. These findings suggest that there is substantial burden of typhoid even in periurban and rural areas outside of Kathmandu.

Over the past 20 years, virtually all of the prospective enteric fever studies in Nepal have been conducted in Lalitpur, a metropolitan city in the Kathmandu Valley [4–7]. The placebo arm of the recent TCV trial conducted in this community demonstrated a high incidence (428 per 100 000 PY) among children 6 months to 15 years of age [18]. Population-based data outside of this community are lacking. One recent study found that cases of clinically diagnosed enteric fever reported through the national Health Management Information System had grown over the past 15 years, reaching reported rates of 1800 per 100 000 PY, highest in the rural areas [11]. However, in prospective surveillance at sites with blood culture capacity, only 4.1% of those clinically diagnosed with typhoid had positive blood cultures for typhoidal *Salmonella,* and this rate ranged between 0 and 2.8% in rural areas of Nepal [11]. Culture positivity in that study was strongly, positively associated with population density. However, the earlier study included fever patients from all rural areas in the catchment of Dhulikhel Hospital. Within the more limited catchment population of the present study, we found no consistent differences in typhoid incidence between the urban (Kathmandu), periurban (Banepa, Dhulikhel) and rural (Paanchkhal, Panauti) communities. In other settings, some studies have shown higher incidence in urban than rural areas [19, 20], whereas others have found the converse [21].

The study catchment area for the periurban and rural area communities was selected based on where enteric fever cases had been detected in the previous 2 years, which biased toward selection of higher-risk communities. Furthermore, Nepal has highly diverse ecosystems, from subtropical communities in the South to alpine villages in the Himalaya. The communities selected for this study were all in 1 province and are not likely to be representative of the typhoid risk in areas throughout the country. Nevertheless, this study adds to what was a sparse collection of population-based data on enteric fever in the country. A major obstacle to generating such data is the resource-intensive nature of population-based surveillance systems; emerging approaches including seroepidemiology and environmental surveillance may enable more efficient typhoid risk mapping in resource-constrained settings [22].

We observed moderate spatial heterogeneity in febrile illness in both catchment areas, with overall higher rates of fever in the rural communities. As anticipated, care seeking for febrile illness at study sites in both communities was strongly predicted by road distance from the site. We found that most of this effect occurred at smaller distances, and that after a point, distance did not further affect the probability of seeking care at the site. Understanding these heterogeneities can be important to the design and interpretation of surveillance approaches.

The median age of enteric fever cases in our study was 20 years, and nearly half of all cases occurred among individuals between the ages of 16 and 25 years. This age distribution is higher than reported in some neighboring South Asian countries, but similar to what has been reported in Lalitpur (median ages of 16 and 20 years for typhoid and paratyphoid, respectively) [4]. In retrospective data reported from Nepal and India for phase 1 of the SEAP study, the median age of typhoid was 19 years in Nepal and 24 years in India [23]. In these settings, catch-up vaccination campaigns among older children, adolescents, and young adults may be important for addressing the burden of typhoid and preventing transmission.

These results should be interpreted within the context of the limitations of the study design and available data. We used healthcare utilization data to adjust for the proportion of patients with enteric fever who did not seek care at the study site; it is possible that individuals who sought care at the study site differed from those who did in ways that were related to their risk of typhoid. One approach to handle this confounding is inverse probability weighting based on household characteristics; while we performed this approach for the overall area estimates, we were unable to do this at finer geographical resolution due to lack of power. We made estimates based on administrative boundaries (wards, municipalities), because this address information was available for all participants enrolled in clinical surveillance. There are no conventional street addresses in Nepal, which precluded more precise geocoding of participants based on the available data. Our estimates are based on the participant’s household address, but typhoid may be acquired outside the household, so the geospatial estimates may not reflect where transmission occurs. We assumed a fixed blood culture sensitivity for all participants based on a previous meta-analysis [17]; in reality, this may vary by age, blood volume, and prior antibiotic use. We opted for more parsimonious and data-driven adjustments to our incidence model.

In conclusion, we found a high incidence of typhoid fever in urban, periurban, and rural communities in and nearby to Kathmandu, with no clear relationship between typhoid incidence and population density. The incidence in all communities was above previously proposed thresholds at which typhoid vaccination would be deemed cost-effective [24]. These findings support the introduction of TCV in Nepal, alongside improved water and sanitation interventions, to prevent both typhoid and paratyphoid fever in communities throughout the country.

## Notes

***Acknowledgments.*** The authors are grateful for the efforts of the Surveillance for Enteric Fever in Asia Project (SEAP) Nepal research assistants, field team, laboratory staff, and laboratory network teams.

***Financial support.*** This work was supported by the Bill & Melinda Gates Foundation (BMGF) (award number OPP1113007).

***Supplement sponsorship.*** This supplement is sponsored by the Sabin Vaccine Institute and made possible by a grant from the Bill & Melinda Gates Foundation.

***Potential conflicts of interest.*** The authors all acknowledge grant support from BMGF. K. D. reports a conditional gift agreement between the Sabin Vaccine Institute (primary grantee for the SEAP grant from BMGF) and the CDC Foundation. S. S. reports grants from the World Health Organization (WHO) during the conduct of the study, and grants from the WHO, BMGF, GlaxoSmithKline, Pfizer, Sanofi Pasteur, and Edinburgh University, outside the submitted work. All other authors report no potential conflicts of interest. All authors have submitted the ICMJE Form for Disclosure of Potential Conflicts of Interest. Conflicts that the editors consider relevant to the content of the manuscript have been disclosed.
